# Therapeutic Outcomes of Biomodulation With Radio Electric Asymmetric Conveyer (REAC) Technology in an 80-Year-Old Female: A Case Report on Anti-cellulite, Circulatory, and Metabolic Optimization Treatments

**DOI:** 10.7759/cureus.72194

**Published:** 2024-10-23

**Authors:** Ludmilla C Higino Rocha, Vania Fontani, Salvatore Rinaldi

**Affiliations:** 1 Brazilian Branch, International Scientific Society of Neuro Psycho Physical Optimization with REAC Technology, São Paulo, BRA; 2 Research Department, Rinaldi Fontani Foundation, Florence, ITA; 3 Department of Regenerative Medicine, Rinaldi Fontani Institute, Florence, ITA

**Keywords:** anti-cellulite treatment, cellular bioelectrical modulation, chronic edema management, circulatory optimization, elderly patient rehabilitation, metabolic optimization, reac

## Abstract

This case report examines the therapeutic outcomes of Radio Electric Asymmetric Conveyer (REAC) anti-cellulite treatment (ACT), circulatory optimization (CO), and metabolic optimization (MO) in an 80-year-old female with multiple chronic conditions, including morbid obesity, type 2 diabetes mellitus, hypertension, and a history of deep vein thrombosis. Each treatment protocol was administered in 18-session cycles. The patient experienced substantial improvements in edema, erythema, pain, and mobility, with a reduced reliance on analgesics. Moreover, there were significant enhancements in sleep quality, mood, mental clarity, and a 7-kg weight loss. These results suggest that REAC ACT, CO, and MO treatments effectively addressed not only localized symptoms but also contributed to broader systemic health improvements by promoting cellular homeostasis and energy production. This case highlights the potential of these REAC treatments as non-invasive and effective options for elderly patients with complex health profiles, encouraging further studies to establish standardized treatment protocols.

## Introduction

This case report presents the therapeutic outcomes of an 80-year-old female patient with multiple chronic conditions who underwent treatment using endogenous biomodulation through the Radio Electric Asymmetric Conveyer (REAC) technology. REAC technology has been extensively studied in the field of endogenous biomodulation, where its ability to restore cellular bioelectric activity and promote physiological balance has been demonstrated [1-3]. Among its applications, REAC technology has shown significant promise in the field of restorative medicine, specifically through the REAC restorative protocol (RPR), which targets tissue repair, inflammation reduction, and metabolic optimization (MO).

The treatments used in this case report anti-cellulite treatment (ACT), circulatory optimization (CO), and MO are integral components of the REAC RPR treatments [4-7]. These protocols were selected to address the patient’s complex medical history, which included morbid obesity, type 2 diabetes mellitus, hypertension, and chronic venous insufficiency. The REAC ACT was aimed at reducing chronic inflammation and improving microcirculation; the CO focused on enhancing vascular health and tissue perfusion, and the MO protocol was designed to optimize metabolic efficiency and energy production. Together, these treatments formed a comprehensive, non-invasive approach to managing both localized and systemic health issues, with the goal of promoting long-term restorative outcomes.

## Case presentation

The patient is an 80-year-old female who presented with multiple chronic health issues and a history of significant comorbidities. Her primary complaints at the time of presentation included persistent edema in the lower limbs, which had been a long-standing concern. Notably, she experienced two episodes of erysipelas in the last two months, both of which were successfully treated with antibiotic therapy. These recurrent episodes indicate a vulnerability to skin infections, likely exacerbated by chronic venous insufficiency and edema.

Her medical history is extensive and includes morbid obesity, which has likely contributed to her other health complications, such as hypertension and type 2 diabetes mellitus, both of which are well-established risk factors for cardiovascular disease. Additionally, she has a history of hypothyroidism, which requires ongoing management and may influence her overall metabolic state. The patient also suffers from chronic insomnia, anxiety, and depression, which have significantly impacted her quality of life and daily functioning.

The patient’s history is further complicated by paroxysmal nocturnal dyspnea, suggesting underlying cardiovascular or respiratory issues. She also has a notable history of pulmonary thromboembolism and has experienced three episodes of deep vein thrombosis (DVT) in the past. Due to this history, she has been on long-term anticoagulant therapy to prevent further thrombotic events. The patient also presents with an asymmetry in the lower limbs that has been present since childhood, indicating a possible congenital or developmental issue. Moreover, she has undergone bilateral hip arthroplasty, suggesting a history of osteoarthritis or another degenerative joint condition.

Her current medication regimen is extensive, reflecting the complexity of her health status. It includes enalapril 20 mg twice daily for hypertension, indapamide SR 1.5 mg as a diuretic, and metformin (Glifage XR) 1000 mg for diabetes management. She also takes carbamazepine 20 mg every 8 hours, likely for neuropathic pain or as a mood stabilizer, and risperidone 1 mg for managing symptoms of anxiety or depression. Additionally, she is prescribed furosemide 40 mg and spironolactone 50 mg, both of which are diuretics that aid in managing her edema and hypertension. Despite her complex medical history, the patient has no known drug, food, or environmental allergies, which simplifies the selection of therapeutic interventions.

Overall, the patient’s clinical picture is indicative of a multifaceted health profile, with chronic conditions affecting multiple organ systems. This complexity underscores the need for a comprehensive treatment approach that addresses both her physical and mental health challenges to improve her overall quality of life and functional status.

Clinical findings

**Figure 1:**
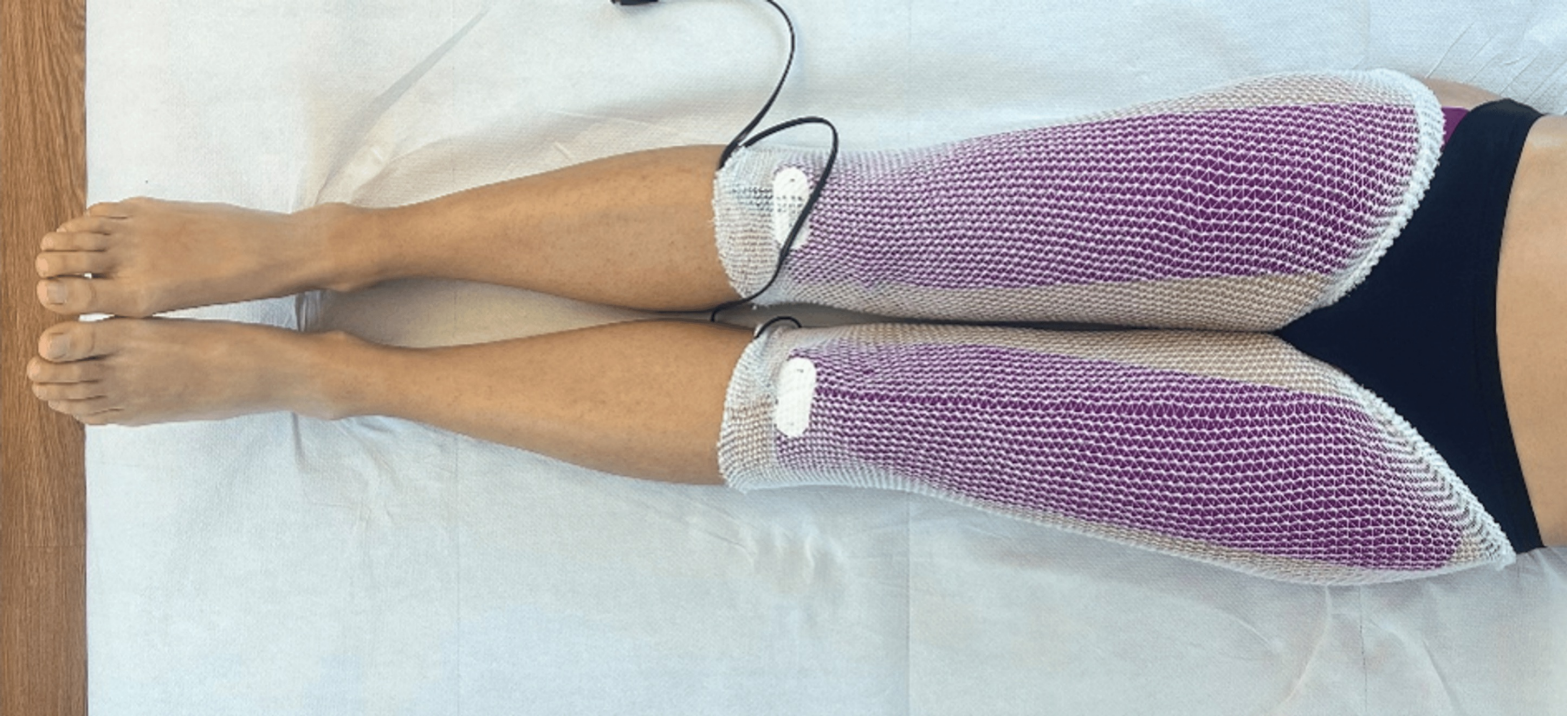
Example of the administration method of REAC ACT, CO, MO treatments The figure shows how the ACPs were positioned on the quadriceps during the treatments. The color of the ACPs is different for ACT, CO, and MO, respectively. REAC: Radio Electric Asymmetric Conveyer; ACT: anti-cellulite treatment; CO: circulatory optimization; MO: metabolic optimization; ACPs: asymmetric conveyer probes

At the time of assessment, the patient presented with chronic edema and erythema in the lower limbs, along with a volume asymmetry in the lower limbs that has been present since childhood (Figure 1). She reported pain in the knees, insomnia, and anxiety.

Therapeutic interventions

The treatment began with the REAC ACT in July 2024. The REAC ACT went beyond addressing the superficial appearance of cellulite by targeting the deeper issue of cellular inflammation. It used the REAC bioelectrical modulation to reduce inflammation, promote tissue repair, and improve microcirculation and lymphatic drainage [1,2]. By addressing the root causes of inflammation [3], REAC ACT not only improved the skin’s appearance but also reduced the underlying processes contributing to cellulite formation. The REAC ACT treatment involved an 18-session cycle, with each session lasting 15 minutes and spaced at intervals of at least one hour, allowing for a maximum of four sessions per day. The treatment was administered by placing the asymmetric conveyor probes, which were interconnected and connected to the BENE 110 device, on the right and left femoral quadriceps (Figure 1).

Follow-up and outcomes

**Figure 2:**
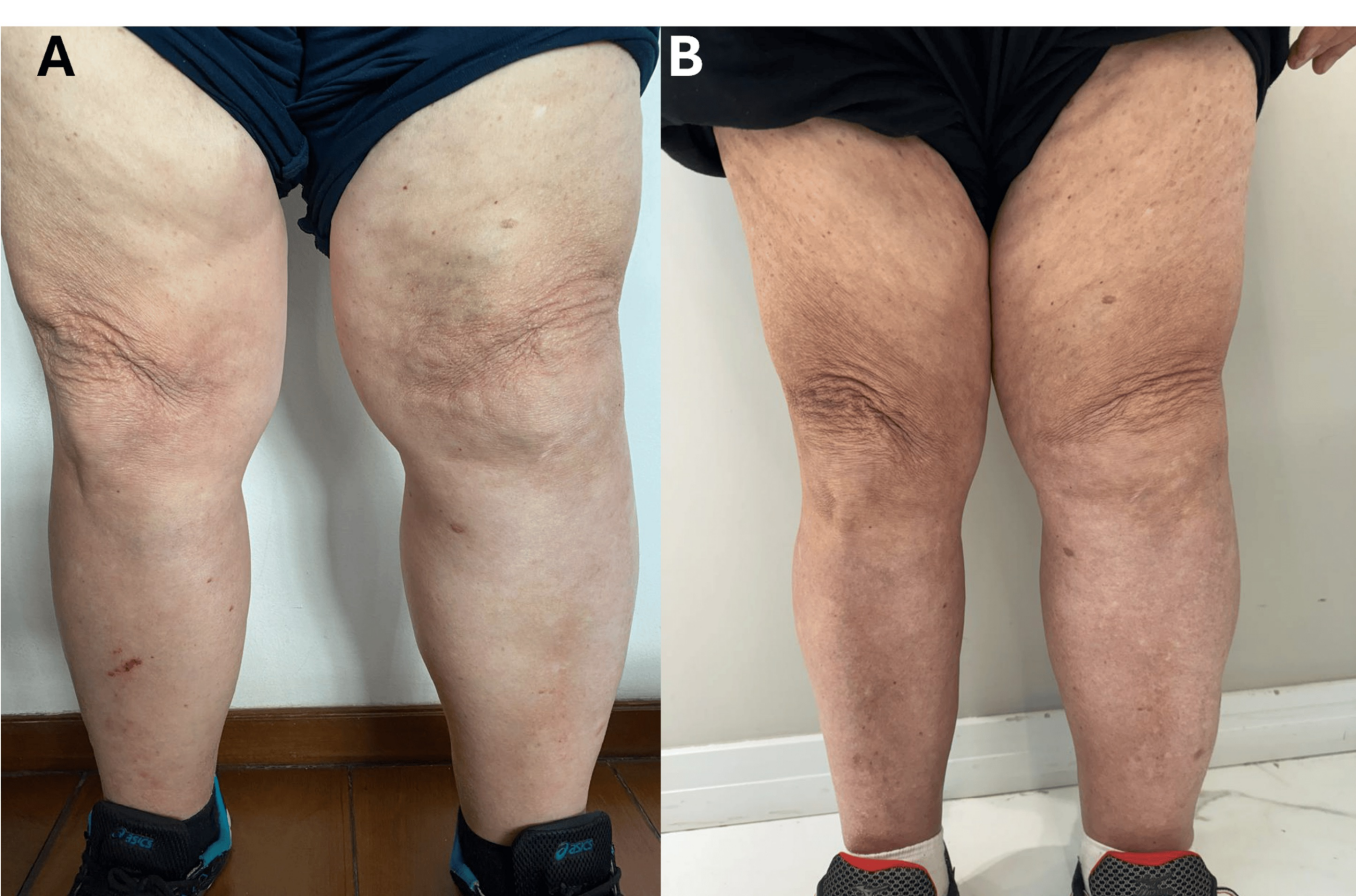
Comparison of the patient’s condition before and after the REAC ACT, CO, and MO treatments A) illustrates the initial condition of the patient’s legs prior to treatment, while B) displays the condition of the legs following the completion of the ACT, CO, and MO treatment cycles. REAC: Radio Electric Asymmetric Conveyer; ACT: anti-cellulite treatment; CO: circulatory optimization; MO: metabolic optimization

At follow-up, the patient demonstrated a significant reduction in both edema and erythema in the lower limbs, suggesting a substantial improvement in her inflammatory status (Figures 2A-2B).

Notably, the patient experienced a weight loss of 7 kg, which indicates an improvement in her metabolic state, likely influenced by the REAC MO treatment. This weight loss contributed to an overall enhancement in the patient’s physical function and mobility.

In addition to these physical changes, the patient showed a marked improvement in vitality, mood, and overall quality of life. She reported feeling more energetic, which positively affected her ability to perform daily activities. This enhancement in vitality was accompanied by a noticeable reduction in pain levels, particularly in the lower limbs, allowing the patient to rely less on analgesic medications.

Objective measurements further confirmed these therapeutic benefits, showing a reduction in the circumference of both the lower limbs and abdominal region. These findings indicate decreased fluid retention and inflammation, as well as possible improvements in fat distribution and muscle tone. Collectively, these outcomes reflect the effectiveness of the REAC ACT, CO, and MO treatments in not only addressing the patient’s primary complaints but also in promoting systemic health and well-being, ultimately contributing to a significant improvement in her overall functional status and quality of life.

## Discussion

The outcomes observed in this case align with and extend current knowledge on the therapeutic applications of REAC RPR (restorative protocol) treatments, particularly for addressing chronic inflammation, improving tissue perfusion, and optimizing metabolic processes [4-7]. Similar results have been documented in studies where REAC treatments led to significant reductions in edema, erythema, and pain in conditions related to venous insufficiency [4] and neuroinflammatory disorders [8,9]. In this case, the patient experienced notable improvements in diuresis, mobility, and a decrease in reliance on analgesics, consistent with previous reports that suggest REAC technology’s capacity to promote systemic bioelectrical rebalancing and enhance tissue repair [10-14].

However, there are important differences that distinguish this case. Unlike prior studies predominantly conducted on younger or middle-aged cohorts, this case involves an 80-year-old patient with a complex array of comorbidities, including morbid obesity, type 2 diabetes, hypertension, and a history of DVT. The observed weight loss of 7 kg, combined with improved metabolic function and enhanced vitality, suggests that REAC MO treatments may have broader systemic benefits than previously reported in older populations with metabolic dysregulation. These findings highlight the potential of REAC technology not only to address local symptoms but also to facilitate systemic metabolic improvements, an area less explored in the existing literature.

Cognitive and emotional improvements observed in this patient, such as enhanced mental clarity, mood stabilization, and improved sleep quality, are consistent with findings from studies on REAC treatments in neuropsychiatric disorders, including autism, fibromyalgia, and mood disorders [11,12,15-17]. The reduction in anxiety and depression symptoms, in particular, reinforces the role of REAC technology in neuromodulation, as documented in cases where it effectively improved neuroplasticity and emotional regulation. This systemic effect on the central nervous system could explain the broad-spectrum improvements seen in both the physical and psychological domains in this patient.

Comparatively, the integration of the REAC ACT, CO, and MO protocols demonstrates a more comprehensive therapeutic impact than what has been documented in studies focusing on singular REAC treatments. While individual protocols have been associated with localized improvements in microcirculation and tissue repair, this case suggests that combining these protocols can generate synergistic effects, addressing multiple underlying pathophysiological mechanisms concurrently. The enhanced microcirculation, reduction in limb circumference, and overall improvements in physical function and quality of life are likely due to the multi-dimensional approach of combining REAC ACT, CO, and MO, which merits further investigation.

Nonetheless, it is crucial to acknowledge the limitations of this case report. As a single case study, the findings cannot be generalized without further validation through controlled clinical trials. Larger, randomized studies with longer follow-up periods are essential to establish the reproducibility and long-term efficacy of REAC treatments, particularly in elderly and multimorbid patient populations. While previous studies have started to shed light on the bioelectrical and cellular mechanisms of REAC technology, future clinical research is needed to rigorously validate its therapeutic potential. This will enable broader adoption of REAC treatments in clinical practice, particularly for patients with complex health conditions.

## Conclusions

The REAC technology, through its ACT, CO, and MO treatment protocols, demonstrated significant therapeutic benefits in this elderly female patient with multiple chronic conditions. The treatments effectively reduced chronic edema, erythema, and pain, improved sleep quality, mental clarity, and overall vitality, and facilitated weight loss and improved metabolic health. These results suggest that REAC ACT, CO, and MO treatments offer a comprehensive, non-invasive therapeutic option for patients with complex health profiles, enhancing both physical and mental well-being.

Nevertheless, the findings of this single case report, while promising, are limited in their generalizability. Larger, well-designed clinical trials are necessary to corroborate these results and explore the long-term sustainability of the observed benefits. We also acknowledge the need for more extensive studies to clarify the mechanisms of action underlying REAC technology, which may further substantiate its efficacy in both localized and systemic health improvements. Future research should include control groups and extended follow-up to provide more robust evidence for integrating REAC treatment protocols into routine clinical practice, especially for elderly patients with complex chronic conditions.

## References

[1] Fontani V, Cruciani S, Santaniello S, Rinaldi S, Maioli M (2023). Impact of REAC regenerative endogenous bioelectrical cell reprogramming on MCF7 breast cancer cells. J Pers Med.

[2] Maioli M, Rinaldi S, Pigliaru G (2016). REAC technology and hyaluron synthase 2, an interesting network to slow down stem cell senescence. Sci Rep.

[3] Maioli M, Rinaldi S, Cruciani S (2022). Antisenescence effect of REAC biomodulation to counteract the evolution of myelodysplastic syndrome. Physiol Res.

[4] Elio C, Fontani V, Rinaldi S, Gasbarro V (2020). REAC-induced endogenous bioelectric currents in the treatment of venous ulcers: A three-arm randomized controlled prospective study. Acta dermatovenerologic.

[5] Fontani V, Coelho Pereira JA, Rinaldi S (2022). Radio electric asymmetric conveyer tissue reparative treatment on post-surgical breast skin necrosis. A report of four cases. Cureus.

[6] Castagna A, Fontani V, Rinaldi S (2022). Radio electric asymmetric conveyer reparative effects on muscle injuries: A report of two cases. Cureus.

[7] Fontani V, Coelho Pereira JA, Carréra Bittencourt M, Rinaldi S (2022). Radio electric asymmetric conveyer (REAC) reparative effects on pressure ulcer (PU) and burn injury (BI): A report of two cases. Cureus.

[8] Panaro MA, Aloisi A, Nicolardi G (2018). Radio electric asymmetric conveyer technology modulates neuroinflammation in a mouse model of neurodegeneration. Neurosci Bull.

[9] Lorenzini L, Giuliani A, Sivilia S (2016). REAC technology modifies pathological neuroinflammation and motor behaviour in an Alzheimer's disease mouse model. Sci Rep.

[10] Rinaldi A, Marins Martins MC, De Almeida Martins Oliveira AC, Rinaldi S, Fontani V (2023). Improving functional abilities in children and adolescents with autism spectrum disorder using non-invasive reac neuro psycho physical optimization treatments: A PEDI-CAT study. J Pers Med.

[11] Rinaldi C, Landre CB, Volpe MI (2023). Improving functional capacity and quality of life in Parkinson's disease patients through REAC neuromodulation treatments for mood and behavioral disorders. J Pers Med.

[12] André Nogueira JA, Souza Bulle Oliveira A, Pereira Motta M (2024). Neurobiological modulation with REAC technology: enhancing pain, depression, anxiety, stress, and quality of life in post-polio syndrome subjects. Sci Rep.

[13] Olivieri EB, Vecchiato C, Ignaccolo N (2011). Radioelectric brain stimulation in the treatment of generalized anxiety disorder with comorbid major depression in a psychiatric hospital: A pilot study. Neuropsychiatr Dis Treat.

[14] Pinheiro Barcessat AR, Nolli Bittencourt M, Duarte Ferreira L, de Souza Neri E, Coelho Pereira JA, Bechelli F, Rinaldi A (2020). REAC cervicobrachial neuromodulation treatment of depression, anxiety, and stress during the COVID-19 pandemic. Psychol Res Behav Manag.

[15] Rinaldi A, Martins MC, Maioli M, Rinaldi S, Fontani V (2023). REAC noninvasive neurobiological stimulation in autism spectrum disorder for alleviating stress impact. Adv Neurodev Disord.

[16] Silva A, Barcessat AR, Gonçalves R (2023). REAC neurobiological modulation as a precision medicine treatment for fibromyalgia. J Pers Med.

[17] Pereira Motta M, Oliveira ASB, André Nogueira JA (2024). Efficacy of REAC neurobiological optimization treatments in post-Polio syndrome: A manual muscle testing evaluation. Journal of Personalized Medicine.

